# Reimbursement of hormonal contraceptives and the frequency of induced abortion among teenagers in Sweden

**DOI:** 10.1186/1471-2458-14-523

**Published:** 2014-05-29

**Authors:** Adam Sydsjö, Gunilla Sydsjö, Marie Bladh, Ann Josefsson

**Affiliations:** 1 Department of Obstetrics and Gynecology, Linköping University, Linköping, Sweden; 2 Department of Clinical and Experimental Medicine, Linköping University, Linköping, Sweden

**Keywords:** Contraceptives hormonal, Induced abortion, Reimbursement, Teenagers

## Abstract

**Background:**

Reduction in costs of hormonal contraceptives is often proposed to reduce rates of induced abortion among young women. This study investigates the relationship between rates of induced abortion and reimbursement of dispensed hormonal contraceptives among young women in Sweden. Comparisons are made with the Nordic countries Finland, Norway and Denmark.

**Methods:**

Official statistics on induced abortion and numbers of prescribed and dispensed hormonal contraceptives presented as “Defined Daily Dose/thousand women” (DDD/T) aged 15-19 years were compiled and related to levels of reimbursement in all Swedish counties by using public official data. The Swedish numbers of induced abortion were compared to those of Finland, Norway and Denmark. The main outcome measure was rates of induced abortion and DDD/T.

**Results:**

No correlation was observed between rates of abortion and reimbursement among Swedish counties. Nor was any correlation found between sales of hormonal contraceptives and the rates of abortion. In a Nordic perspective, Finland and Denmark, which have no reimbursement at all, and Norway all have lower rates of induced abortion than Sweden.

**Conclusions:**

Reimbursement does not seem to be enough in order to reduce rates of induced abortion. Evidently, other factors such as attitudes, education, religion, tradition or cultural differences in each of Swedish counties as well as in the Nordic countries may be of importance. A more innovative approach is needed in order to facilitate safe sex and to protect young women from unwanted pregnancies.

## Background

Swedish abortion laws are among the most liberal in the Nordic countries as well as most western countries. Since 1975 induced abortion on demand by the pregnant woman is legal up to the 18^th^ gestational week of the actual pregnancy [1]. Rates of induced abortions have remained more or less unchanged since 1975 in contrast to the other Nordic countries, in spite of a public Swedish family planning program which is free of charge [2].

An induced abortion is not totally harmless from a psychological aspect [3] and may lead to medical complications, albeit few, and may interfere with future planned parenthood [3].

The high present rates of induced abortions in Sweden, and especially among teenagers in comparison to other western societies have raised concern and several strategies have been proposed by studies [4]. An effect of the findings in studies on this subject has often been to propose easily available and subsidized hormonal contraceptives for young women [5-7].

Although the Swedish Parliament regulates laws concerning induced abortion, the implementation of the laws and provision of family planning centers are decided by each Swedish county separately. For that reason, there are differences in the amount of reimbursement of for instance hormonal contraceptives between the counties. The upper age limit for reimbursement of contraceptives also differs between the counties.

In a recent study investigating the opinion of county council politicians, on attitudes towards abortion and the present abortion rates, the main suggestion from the politicians in order to reduce the number of unwanted pregnancies and hence the number of induced abortions, were to increase the reimbursement and thus make hormonal contraceptives easier available to young women [8].

As the reimbursement of hormonal contraceptives differs between the counties in Sweden one should expect rates of induced abortions to be lower where reimbursement is high. Therefore, it is of interest to study whether costs for contraceptives are a main deciding factor and has an impact on the rates of induced abortions. The primary aim of this study was to investigate the relation between induced abortions and reimbursement of dispensed hormonal contraceptives in Sweden among young women. Secondary aim was to make comparisons with the Nordic countries Finland, Norway and Denmark.

## Methods

Information on induced abortions, prescribed and dispensed hormonal contraceptives and the relation between counties with regard to induced abortion are all available in public official statistics provided.

We have obtained data on drugs having the Anatomical Therapeutic Chemical code G03A that includes all combined hormonal contraceptives except the vaginal ring and progestin-only contraceptives.

The amount of hormonal contraceptives is shown as “Defined Daily Dose/thousand women” (DDD/T) of G03A as presented by the Swedish Medical Board on Health and Welfare [9] and is presented per 1000 women aged 15-19 years. DDD/T is considered to be a useful mean to compare differences in the use of sold medical products. We included women 15-19 years of age, even though some counties subsidize the costs for contraceptives up to the age of 24. By inclusion of women under 19 years of age it was possible to compare all counties in Sweden.

The data in this study on induced abortion in the Nordic countries were derived from official statistics as presented in a joint venture by the Nordic governments (Denmark, Norway, Sweden and Finland) and health care providers, STAKES [10]. We have chosen to omit Iceland in this study, as the population of Iceland is merely about 300 000 inhabitants.

Statistical analysis is limited to Spearman’s correlation (rho). All tests were two-sided and a p-value <0.05 was considered statistically significant.

Ethical approval has not been asked for since the study is based on aggregated numbers from official statistics.

## Results

**Figure 1:**
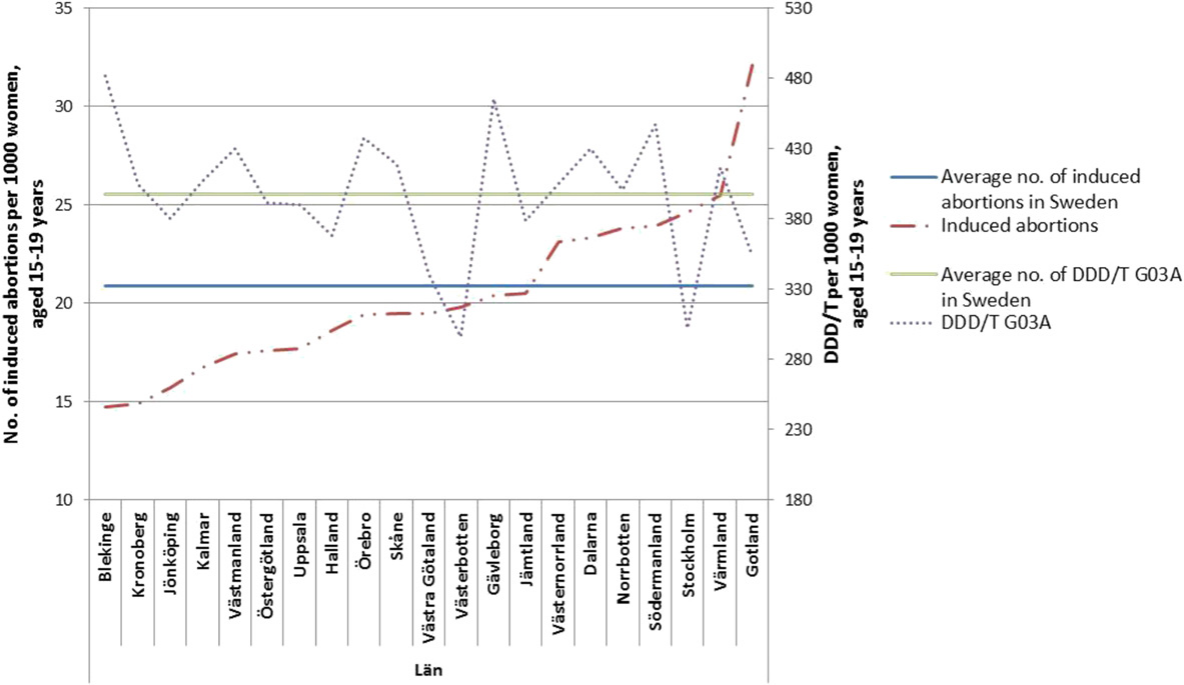
Induced abortion in all Swedish counties in relation to DDD/T in 2010.

**Figure 2:**
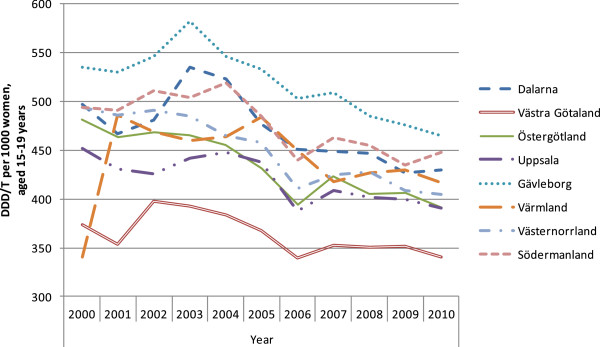
Hormonal contraceptives presented as DDD/T in eight counties with the same subventions 2000-2010.

**Figure 3:**
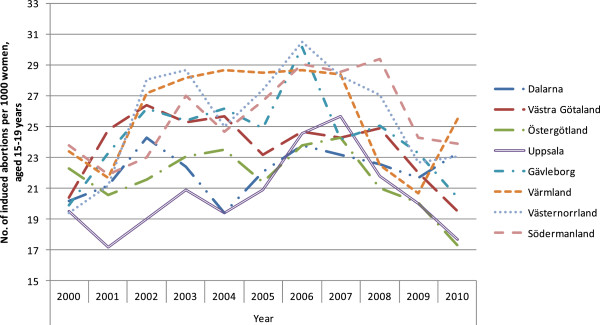
**Induced abortion in the same counties as in Figure **2**, 2000-2010.**

**Figure 4:**
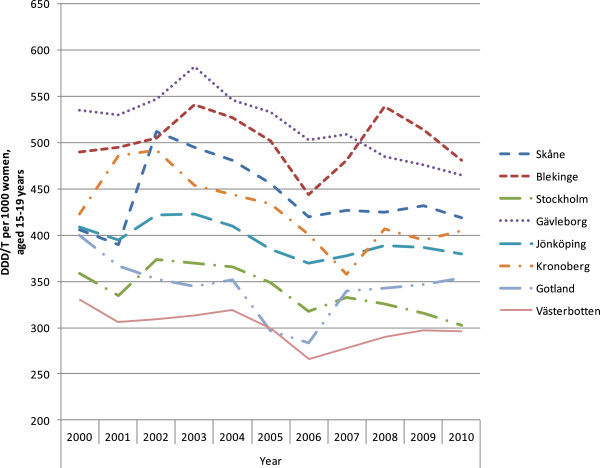
**Sales of G0A3 expressed in DDD/T in eight counties, Skane and Blekinge reimburse all costs.** Vasterbotten does not reimburse at all. The other 5 counties reimburse to some extent.

**Figure 5:**
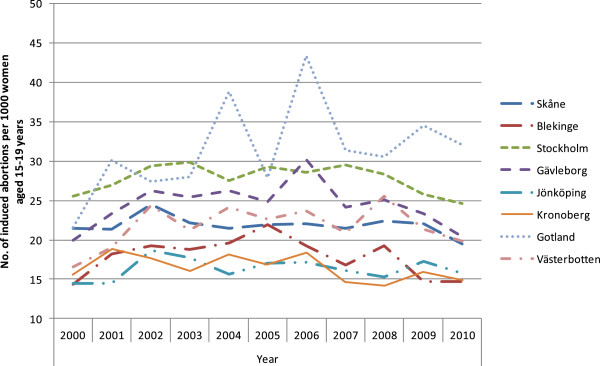
**Differences in induced abortion in the same eight counties as in Figure **4**.**

**Figure 6:**
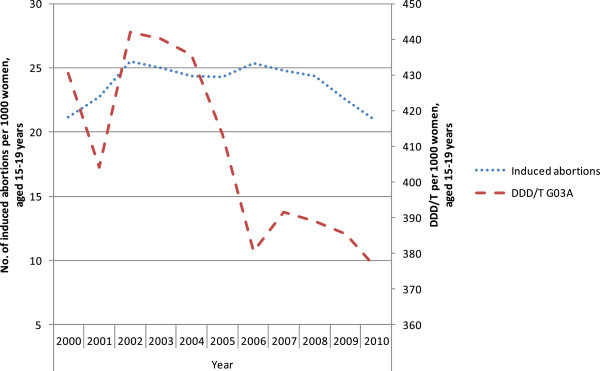
Induced abortion and dispensed hormonal contraceptives 2000-2010 in all counties in Sweden.

**Figure 7:**
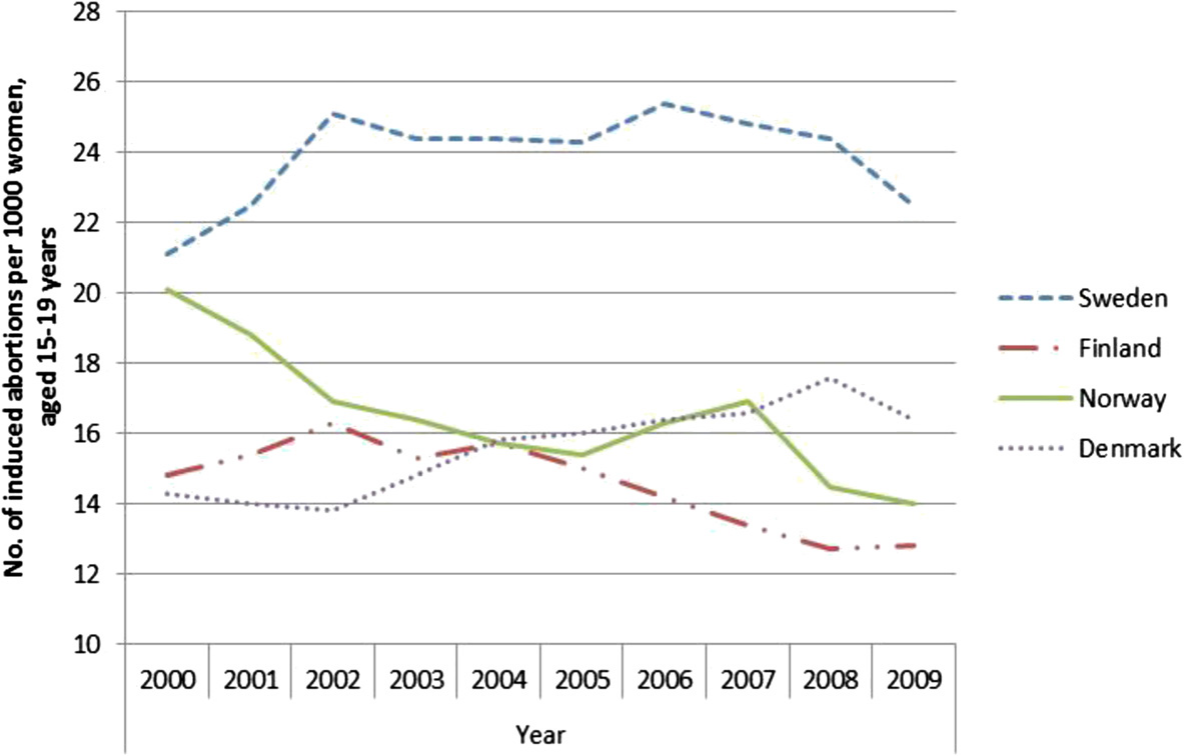
Induced abortion among women 15-19 years in Nordic countries 2000-2009.

The results are presented in seven figures.Figure 1 combines prescribed and dispensed hormonal contraceptives presented as DDD/T per 1000 women 15-19 years old and number of induced abortions for all counties in Sweden in 2010. No correlation between abortion rates and dispensed hormonal contraceptives was observed during the year of 2010 (rho = -0.158 p = 0.493).Figures 2 and 3 show differences in DDD/T per 1000 women 15-19 years old and differences in rates of induced abortions for eight Swedish counties over a time period of 10 years, 2000-2010. The counties chosen have one factor in common; the reimbursement makes the hormonal contraceptives equally expensive for the woman. From the figures it derives, that both sold hormonal contraceptives and the rates of induced abortions differ between the eight Swedish counties. There was no statistically significant correlation between DDD/T per 1000 women and rates of induced abortions in any of the eight counties (all p-values >0.05).Figures 4 and 5 show counties with different amount of reimbursement. The counties Skane and Blekinge are offering hormonal contraception for free while the county Vasterbotten has no reimbursement at all. No apparent correlation was found in this comparison either. All correlations between DDD/T per 1000 women and rate of induced abortion had p-values >0.05 except for county of Skane where rho = 0.615 and p = 0.044 indicating a positive relationship between DDD/T and rate of induced abortion. That is, the more DDD/T that was sold the higher the rate of induced abortion.In Figure 6, mean values for induced abortions and DDD/T for women 15-19 years old for the period 2000-2010 are presented. A drop in sales was observed from 2004 and onwards, but it was not followed by any increase in induced abortion. Instead, the rates of induced abortion also declined.Figure 7 shows the development of induced abortion in the Nordic setting 2000-2009. Notable, Finland and Denmark have no reimbursement at all for hormonal contraceptives yet the developments of the rates of induced abortions are quite different. From 2004 and onwards Finland has had a steady drop in number of induced abortions while in Denmark the number of induced abortions have increased in the same time period.

## Discussion and conclusions

Although this study presents results compiled from official statistics in Sweden, the result may be of interest as a common assumption, not only in Sweden, is that reimbursement of hormonal contraceptives will lead to a decrease in the rate of induced abortion among the youngest women [8,11,12]. However, no clear connection is found in this paper between actual sales of hormonal contraceptives (GO3A), induced abortion or the relation between different counties with different amount of reimbursement among young women 15-19 years. Our study suggests that in a modern welfare society there is neither a straight agreement between induced abortion and the amount of prescribed and dispensed hormonal contraceptives, nor reimbursement and rates of induced abortion, not only in Sweden, but also, in comparison with other Nordic countries.

Introducing subsidized contraceptives has generally been supposed to work towards lowered rates of induced abortion and in the Swedish county of Gavle a program including reimbursement was introduced in 1989. Most Swedish counties have followed this program with the exception of Vasterbotten. However, there are differences in the amount of reimbursement where some counties even reimburse the whole cost of hormonal contraceptives (e.g. Skane and Blekinge). Studies after the start of the program in the county of Gavle showed a considerable decrease in teenage abortions (50%) and an increase in the use of the oral contraceptives (60%). But, when rates of abortion among young women, 15-19 years, are followed from 1989 up to now, this encouraging result is not sustained. Instead, it seems more to follow the national abortion trends in this age group in Sweden and was at 2010, rather high in comparison with other Swedish counties [5,6].

Recently, due to changes in the support in the county of Stockholm, worries have been put forward. By not including the newest and most expensive types of contraceptive pills in the reimbursement system, there has been a worry that this would lead to a fall in the use of hormonal contraceptives. A fall in dispensed hormonal contraceptives is indeed observed, but this is not paralleled to a rise in the numbers of induced abortion, which declined as in the rest of Sweden [7].

Our paper is to a certain degree hampered by the fact that although a county might contribute to cover the costs, there are slight differences regarding which different hormonal contraceptive products that are included in the reimbursement. Some often prescribed and popular products have recently been removed from the reimbursement by the pharmaceutical companies themselves, e.g. in Stockholm county. However, generic products are still available. These changes have evidently not increased rates of induced abortion in the county of Stockholm.

Different approaches in order to reduce the abortion rates have been implemented in the Nordic countries and this may result in low figures compared to Sweden [2]. In Norway, a reduction in cost for hormonal contraceptives among the youngest women led to a considerable lower rate of abortion. Nevertheless, the rate of induced abortion in Norway is in parity with Denmark, a country with no reimbursement at all in this aspect [10]. Neither has Finland any reimbursement of hormonal contraceptives. All Nordic countries are regarded as modern well fare states and do not lack founds for providing a good health care for their inhabitants. Evidently, comparisons between different societies indicate, that different amounts of reimbursement is not the sole explanation to differences in rates of induced abortion. To study what must be considered as reasonable proportions or changes in rates of induced abortions, straight comparisons between societies with similar family planning programs may be of help. The Nordic countries seem ideal, as they have so much in common e.g. health care, religion and culture. Support for families in these countries, for instance in connection with pregnancy and childbirth, is well recognized.

In this study, we did not try to estimate the impact of use of non-hormonal contraceptives. It is reported, that for instance the use of condoms is very often practiced in Finland, and such tradition may partly explain differences observed. However, condoms are easily available also in Sweden and obtainable and distributed through the Youth clinics free of charge for the youngest group of teenagers.

Maybe family planning in countries such as Denmark and Finland is more informative on the risks taken in relation to future fertility, which certainly is relevant to have acquired knowledge on. Also it might be suspected, that although family planning in Sweden is similar all over the country, it may be influenced by a counties local traditions or cultural differences. An interesting question in this context is also which information is given to young people on sexually transmitted diseases, which is relatively frequent among young people. Thus, by using condoms as a rule, this is avoided, but also protects against unwanted pregnancy [13,14].

Induced abortion, both among the youngest (≤19 years) as well as among all women (15-44 years), has marked variations over time [2]. It is a common observation in studies that use of hormonal contraceptives is subjected to changes due to alarming reports on possible complications like for instance venous thromboses [15]. Such complications are well recognized, but a new “case report” in the media may cause a drop in the use of hormonal contraceptives, which may lead to an increase in the rate of induced abortion. Our study, however, indicates that maybe this is not the sole explanation, as we found no strict correlation between DDD/T of hormonal contraceptives and induced abortion in this age group. A drop in DDD/T does not necessarily equal a rise in rates of abortion.

The difference in Nordic countries may be caused by different traditions in the use of non-hormonal contraceptives and also at which age when a young girl starts to be sexually active. One explanation thus might be that young people start their sexual life at different ages in different counties in Sweden, depending on, religion, tradition or other cultural factors, for instance, differences between urban areas as compared to more rural areas.

In a recent study on politicians knowledge and opinions on induced abortion performed in 3 counties in southeast Sweden, we found, that in spite the obvious fact, that although this issue was considered as a “women’s right” question, it was considered a difficult topic to discuss, as the politicians were afraid to be labeled as conservative [8]. For that reason, in Sweden it is not allowed to keep a national register on induced abortion, even if such a register would provide useful information. Such ambiguity indicates, that in spite of liberal laws, the society still consider induced abortion as a partly moral or ethical question.

In this study, based on official statistics, we did not find any clear correlation between the rates of induced abortion and dispensed hormonal contraceptives in the youngest group of women, 15-19 years, and reimbursement in all 21 counties of Sweden. Evidently, other factors such as attitudes, education, religion, tradition or cultural differences in each Swedish county as well as between the Nordic countries may be considered of importance. Our conclusion is that reimbursement is not enough. A more innovative approach is needed in order to facilitate safe sex and to protect young women from unwanted pregnancies.

## Competing interests

The authors declare that they have no competing interests.

## Authors’ contributions

AS, designed the study and was responsible for analyzing and prepared the manuscript, GS and AJ analyzed and drafted the manuscript, MB did the statistical work and prepared the manuscript. All authors read and approved the final manuscript.

## Pre-publication history

The pre-publication history for this paper can be accessed here:

http://www.biomedcentral.com/1471-2458/14/523/prepub
